# Self-monitoring and self-management of oral anticoagulation

**DOI:** 10.1590/S1516-31802010000400015

**Published:** 2010-07-01

## Abstract

**BACKGROUND:**

The introduction of portable monitors (point-of-care devices) for the management of patients on oral anticoagulation allows self-testing by the patient at home. Patients who self-test can either adjust their medication according to a pre-determined dose-INR schedule (self-management) or they can call a clinic to be told the appropriate dose adjustment (self-monitoring). Several trials of self-monitoring of oral anticoagulant therapy suggest this may be equal to or better than standard monitoring.

**OBJECTIVES:**

To evaluate the effects of self-monitoring or self-management of oral anticoagulant therapy compared to standard monitoring.

**SEARCH STRATEGY:**

We searched the Cochrane Central Register of Controlled Trials (CENTRAL) (*The Cochrane Library* 2007, Issue 4), MEDLINE, EMBASE and CINAHL (to November 2007). We checked bibliographies and contacted manufacturers and authors of relevant studies. No language restrictions were applied.

**SELECTION CRITERIA:**

Outcomes analysed were thromboembolic events, mortality, major haemorrhage, minor haemorrhage, tests in therapeutic range, frequency of testing, and feasibility of self-monitoring and self-management.

**DATA COLLECTION AND ANALYSIS:**

The review authors independently extracted data. We used a fixed-effect model with the Mantzel-Haenzel method to calculate the pooled risk ratio (RR) and Peto’s method to verify the results for uncommon outcomes. We examined heterogeneity amongst studies with the Chi^2^ and I^2^ statistics.

**MAIN RESULTS:**

We identified 18 randomized trials (4723 participants). Pooled estimates showed significant reductions in both thromboembolic events (RR 0.50, 95% CI 0.36 to 0.69) and all-cause mortality (RR 0.64, 95% CI 0.46 to 0.89). This reduction in mortality remained significant after the removal of low-quality studies (RR 0.65, 95% CI 0.46 to 0.90). Trials of self-management alone showed significant reductions in thromboembolic events (RR 0.47, 95% CI 0.31 to 0.70) and all-cause mortality (RR 0.55, 95% CI 0.36 to 0.84); self-monitoring did not (thrombotic events RR 0.57, 95% CI 0.32 to 1.00; mortality RR 0.84, 95% CI 0.50 to 1.41). Self-monitoring significantly reduced major haemorrhages (RR 0.56, 95% CI 0.35 to 0.91) whilst self-management did not (RR 1.12, 95% CI 0.78 to 1.61). Twelve trials reported improvements in the percentage of mean INR measurements in the therapeutic range. No heterogeneity was identified in any of these comparisons.

**AUTHORS’ CONCLUSIONS:**

Compared to standard monitoring, patients who self-monitor or self-manage can improve the quality of their oral anticoagulation therapy. The number of thromboembolic events and mortality were decreased without increases in harms. However, self-monitoring or self-management were not feasible for up to half of the patients requiring anticoagulant therapy. Reasons included patient refusal, exclusion by their general practitioner, and inability to complete training.

**FURTHER INFORMATION:** Centro Cochrane do Brasil Rua Pedro de Toledo, 598 Vila Clementino — São Paulo (SP) — Brasil CEP 04039-001Tel. (+55 11) 5579-0469/5575-2970 http://www.centrocochranedobrasil.org.br/

## COMMENTS

Coumarins or vitamin K antagonists are oral anticoagulant drugs with wider use in daily medical practice. Because of their pharmacokinetic and pharmacodynamic characteristics, frequent laboratory monitoring is required, since inappropriate use can result in thrombotic or major and minor hemorrhagic complications. This laboratory monitoring is performed through measurement of the prothrombin time, expressed as the international normalized ratio (INR). It should be done monthly or every 45 days, among stable patients. Portable monitors, which make it possible for patients to self-test, are increasingly being used in developed countries. Therefore, it is important to evaluate the impact of INR monitoring using this equipment, with regard to the frequency of new thrombotic events and bleeding complications, in order to instill confidence among physicians and patients.

This review of 18 studies showed that there were significant reductions in thrombotic events and mortality through the use of portable monitors. Although there was a difference between self-management (self-adjustment of doses) and self-monitoring (self-testing and calling a clinic for the dose to be adjusted) regarding new thromboembolic events and major bleedings, it was possible to show that patients had a better quality of life under oral anticoagulant therapy. Nevertheless, self-testing could not be used by all patients. Moreover, even though several portable monitors are available, with different costs and sensitivities, the authors did not clarify which equipment was used or whether they observed differences between them. However, this review shows that using portable monitors to check the INR is a condition that can alter and improve the clinical evolution of patients under oral anticoagulant treatment. In addition, this review shows that new studies need to be developed in order to evaluate the differences in economic impact between the traditional INR method and portable monitors, and even between the several portable monitors.

